# The Problem with Time: Application of Partial Least Squares Analysis on Time-Frequency Plots to Account for Varying Time Intervals with Applied EEG Data

**DOI:** 10.3390/brainsci15020135

**Published:** 2025-01-30

**Authors:** Jessie M. H. Szostakiwskyj, Filomeno Cortese, Raneen Abdul-Rhaman, Sarah J. Anderson, Amy L. Warren, Rebecca Archer, Emma Read, Kent G. Hecker

**Affiliations:** 1 Faculty of Veterinary Medicine, University of Calgary, Calgary, AB T2N 4Z6, Canada; jmhart@ucalgary.ca (J.M.H.S.); alwarren@ucalgary.ca (A.L.W.); rebecca.archer@ucalgary.ca (R.A.); 2 Department of Community Health Sciences, Cumming School of Medicine, University of Calgary, Calgary, AB T2N 4Z6, Canada; fcortese@ucalgary.ca; 3 Hotchkiss Brain Institute, University of Calgary, Calgary, AB T2N 4Z6, Canada; 4 Department of Pathology and Laboratory Medicine, Faculty of Medicine, University of British Columbia, Vancouver, BC V6T 1Z4, Canada; raneena@mail.ubc.ca; 5 Department of Cell Biology and Anatomy, Cumming School of Medicine, University of Calgary, Calgary, AB T2N 4Z6, Canada; anderssj@ucalgary.ca; 6 College of Veterinary Medicine, Office of Professional Programs, The Ohio State University, Columbus, OH 43210, USA; read.65@osu.edu

**Keywords:** EEG, multivariate, partial least squares, assessment, decision-making

## Abstract

**Background/Objectives:** When attempting to study neurocognitive mechanisms with electroencephalography (EEG) in applied ecologically valid settings, responses to stimuli may differ in time, which presents challenges to traditional EEG averaging methods. In this proof-of-concept paper, we present a method to normalize time over unequal trial lengths while preserving frequency content. **Methods:** Epochs are converted to time-frequency space where they are resampled to contain an equal number of timepoints representing the proportion of trial complete rather than true time. To validate this method, we used EEG data recorded from 8 novices and 4 experts in veterinary medicine while completing decision-making tasks using two question types: multiple-choice and script concordance questions used in veterinary school exams. **Results:** The resulting resampled time-frequency data were analyzed with partial least squares (PLS), a multivariate technique that extracts patterns of data that support a contrast between conditions and groups while controlling for Type I error. We found a significant latent variable representing a difference between question types for experts only. **Conclusions:** Despite within and between subject differences in timing, we found consistent differences between question types in experts in gamma and beta bands that are consistent with changes resulting from increased information load and decision-making. This novel analysis method may be a viable path forward to preserve ecological validity in EEG studies.

## 1. Introduction

When seeking to understand the neurocognitive processes that underscore behavior, ecological validity is often compromised for experimental rigor. For example, decision-making is studied with carefully designed, controlled experiments that attempt to decompose this cognitive process into basic components, such as signal detection seen in motion perception or face/object discrimination tasks [1]. To understand the neural underpinnings of decision-making, researchers use electroencephalography (EEG), functional magnetic resonance imaging (fMRI), or other neuroimaging modalities to examine the exact moment in time when a decision is made, based on a controlled stimulus, averaged over many trials to reduce noise or random effects that exist on any single trial [1].

In real life, decision-making can be complex, context-dependent, and made under varying degrees of time constraint. When people are presented with a problem (e.g., a doctor working through a clinical case, executives making corporate decisions, students taking an examination, etc.) and asked to make a decision, there is natural variation within and between people on when a decision occurs and the time leading up to that decision is invariably important to what the decision is and how it was made. For example, one student may respond to a multiple-choice question in 30 s while another takes 3–4 min to respond to the same question. In the time period between reading a question and providing a response, processes such as signal detection, memory retrieval, and working memory, as well as distraction or mind wandering, may occur continuously with decision-making until the point in time the person feels confident enough in their answer to respond [2]. Herein lies the problem; using current experimental EEG protocols and analyses, we can time lock immediately before and after a decision is made, but in so doing, we lose valuable information about the processes involved in the variable time window leading up to that point.

This is especially true when using EEG, as precision in the time domain is considered an advantage of the technology. So why do we tend to constrain or lock to timed events? The main reason is that it provides an easy way to sift through EEG data to differentiate brain activity signals from noise. Even when measurement error inherent to EEG recording, such as electrical noise in the recording environment, is effectively handled with filtering techniques [3], noise remains because the brain is complex and rarely (if ever) does just one thing at a time [4]. Because this noise is random, if one can generate averages, the true brain activity signal should be revealed.

For example, EEG has been effectively used to study brain activity related to working memory [5,6], emotion classification [7,8] and decision-making [6]. It is common for EEG studies such as these to present a participant with many repetitions of stimuli or prompts in different conditions and/or over different groups of individuals for aggregate comparison. Traditional methods, such as event-related potential (ERP) analysis, take a consistent piece of information, time-locked to a stimulus onset or response, and create averages per subject, group, or condition. Through this approach, the inherent variability in brain data for individual trials is managed by the averaging process, and what remains is thought of as the true signal associated with a stimulus or response, provided enough trials were collected. In the previous examples, Onton et al. [5] used over 100 repetitions, and Jacobs et al. [6] used 576 trials.

Differences in the height or depth of peaks in the characteristic ERP can then be related to differences in cognitive processes. Typically, the more time that passes from the time-locking event, the more difficult it is to find a consistent response. As a result, ERP analyses tend to focus on <400 ms after response. For example, the P300 is consistently observed at the vertex (Cz) around 300 ms after stimulus onset and is related to the improbability of inputs [9], and reward positivity, RewP, appears around the frontocentral region (FCz) as a positive peak between 250 and 350 ms following incorrect responses [10]. In the context of a decision-making study where participants are presented with a written scenario and asked to select a response from a list of possible answers, like a multiple-choice question, if we were to take the ERP approach, we could see the visual impact of the stimulus appearing on screen, or the response components following incorrect responses, but it is unlikely that we could capture reasoning that occurs seconds after stimulus onset and before the response.

In comparison, frequency analysis of EEG data converts the signal from a certain time window around an event into frequency space [11,12,13,14,15,16]. Because time information is lost entirely in the transformation to the frequency domain, this method could be used to compare activity over variable trial lengths. However, if differences were found when frequency information is considered over conditions or groups, it would be unclear whether the differences are driven by the conditions or groups or by changes in spectral power over time that are captured in different-length trials.

In contrast to pure frequency analysis, time-frequency analysis converts the signal into frequency space for small time windows around an event. We can compute the perturbations in the power spectrum (Δ*P*) over a sliding latency window and then average across data trials to obtain time-frequency plots described by:

$$\Delta P\left(f,t\right)=\frac{1}{n}\;\sum_{k=1}^{n}|F_{k}\left(f,t\right){|}^{2}$$

where for *n* trials, $F_{k}\left(f,t\right)$ is the spectral estimate of trial *k* at frequency *f* at time *t* [modified from 17]. This allows for preserving some timing information; as such, it seemingly overcomes the issue of frequency analysis, which is unable to decipher whether differences are due to condition, group, or spectral power over time. Time-frequency analysis has effectively demonstrated the distinction between working memory and decision-making in a memory retrieval task [6]. It is most common to examine similar time ranges as with ERP analysis (<1000 ms) [17]. Unfortunately, like ERP analysis, we still have a time component that can differ from trial to trial, posing difficulty in generating averages.

With both time- and frequency-based methods, generating time-locked averages gives the advantage of focusing the signal on something that is produced consistently due to a given stimuli or response. Given the applied nature of our imaging research, using EEG to study health professions, education learning and decision-making led us to our proof-of-concept idea to address variable time in a manner that would open the opportunity for more ecologically valid EEG experiments. To proceed with creating averages on trials of unequal lengths, we propose a method that allows us to assess the entirety of a trial with variable lengths. In this method, we converted each trial into time-frequency space and resampled the data from unequal-length trials into an equal number of timepoints to reflect the proportion of the trial completed rather than time itself. By converting the data to time-frequency spectral power, we can resample unequal-length time-frequency plots to generate epochs with the same number of timepoints while preserving the frequency content at each timepoint as it was calculated prior to resampling.

Because the aim is to find patterns of times and frequencies that are consistently associated with experimental design but may not follow rigid timelines, multivariate, data-driven methods may provide insight. Independent component analysis (ICA) is a powerful, data-driven method for separating brain signals into statistically independent sources. For example, Onton et al. [5] found certain components that differentiate aspects of working memory using ICA. However, by itself, ICA does not examine group- and task-dependent correlations between brain activity and performance. We suggest that using partial least squares (PLS) provides useful information as it extracts patterns of activity that support a contrast between design elements. PLS is similar to other multivariate techniques, such as principal component analysis (PCA), in that contrasts across conditions or groups typically are not specified in advance; rather, the algorithm extracts latent variables (LVs) in order of the amount of covariance explained (with the LV accounting for the most covariance extracted first). The outcome of PLS analysis, therefore, is a contrast between groups and conditions and the pattern of brain data that stably contributes to the LV. PLS also conducts all comparisons in a single step, and therefore, correction for multiple comparisons is not required. The software PLSGUI (PLSGUI v13: Rotman Research Institute at Baycrest Centre, Toronto, ON, Canada), was developed to utilize PLS analyses to extract spatiotemporal patterns in ERP [18,19], which uses time by channel, two-dimensional data and has been successful in extracting meaningful spatiotemporal patterns [20]. This methodology was expanded to look at multiscale entropy measures (MSE), a measure of meaningful complexity in EEG signal, where the data are still two-dimensional, but instead of timepoints, the second dimension is time scale [21,22]. In the current study, we will be expanding these uses to include 3 dimensions, channel by time by frequency, collapsed into channels x time/frequency. Specifically, we will be assessing spectral power estimates over the proportion of trials complete.

To test our resampling and PLS method, we focused on EEG data collected while participants completed common methods for assessing clinical reasoning in health professions education. Namely, questions in which participants are presented with a written scenario with five possible options to select from (multiple choice questions) and script concordance questions, in which participants are presented with a question stem and a Likert-like scale of agreeability meant to assess higher-order cognitive processing and uncertainty associated with clinical reasoning [23,24]. Variability in decision-making cognitive processes and the time required to complete them are common for these assessment methods; therefore, resampling to represent the percent complete, rather than using time, allows us to capture the entirety of each individual’s cognitive response to the question. To our knowledge, there are no published studies examining brain function during completion of multiple choice and script concordance questions, which are meant to assess decision-making processes in a naturalistic setting using neuroimaging methods such as EEG, and we propose that resampling the data to accommodate time may help further develop this area.

We used EEG data collected from 8 novices (veterinary medicine students near the end of their training) and 4 experts (practicing veterinarians) while performing two different assessment question types (multiple choice and script concordance) written by experts in the field of Veterinary Medicine. We used both univariate (*t*-tests) and multivariate approaches (PLS) [18,19] to determine whether there is any consistency in brain activity that may represent different cognitive processes through the course of a trial that may differ with conditions and groups.

## 2. Materials and Methods

### 2.1. Participants

Novice participants (*n* = 9) were veterinary medicine students near the end of their preclinical training. Expert participants (*n* = 4) were practicing veterinarians with 5–15 years of experience in primary care practice (small and large animals). One novice subject was discarded due to technical difficulties during data recording. Participants confirmed they were able to wear an EEG cap for an hour, had no known neurological conditions, and had to have normal or corrected-to-normal vision. This study was approved by the Conjoint Health Research Ethics Board at the University of Calgary (Ethics ID: REB 17-0788)

### 2.2. Multiple Choice and Script Concordance Questions

**Figure 1:**
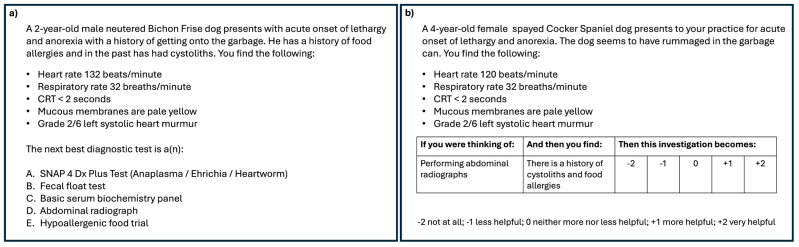
(**a**) Multiple choice question sample; (**b**) Script concordance question sample. Questions are designed to be parallel in content.

Thirty multiple-choice questions that were developed and validated by content experts at the University of Calgary for use in assessing veterinary medicine students’ declarative knowledge were selected for this study. Each question presented a realistic clinical scenario or clinical case with 5 possible answers (Figure 1a). Each question was meant to assess the synthesis or application of declarative knowledge regarding clinical practice to select the most likely diagnosis or treatment. Thirty script concordance questions were developed to match the content of the multiple-choice questions. Each script concordance question presented clinical scenarios or cases similar to those of the multiple-choice questions. However, rather than 5 possible options to select from, proposed diagnoses, investigations or management plans are presented. Participants were then presented with new information and asked how appropriate/suitable the diagnosis/investigation/plan was, considering the new information provided. Participants were asked to respond using a Likert-like scale typically ranging from −2 (very suitable) to +2 (very suitable) (Figure 1b).

### 2.3. Experimental Procedure

Participants were seated in front of a computer screen which displayed the questions. The distance from the screen was 60 cm, and the screen size was 34 × 22 cm, for a visual angle of 31.6° × 20.8°. Questions were presented in a randomized blocked design with either all multiple choice or all script concordance questions first, followed by all the questions for the other question type, with all participants answering all 60 questions (30 multiple choice, 30 script concordance). Questions within each block were presented in random order. A fixation cross was presented prior to each trial for 1000–1800 ms. Participants were instructed to look at the fixation cross, then read the question and respond. Participants were given as much time as they needed to respond to the question on a keyboard in front of them.

### 2.4. EEG Acquisition and Preprocessing

Participants were seated in a sound-attenuated room during the experiment. We recorded continuous EEG data from 32 channels (FP1, FP2, F7, F3, Fz, F4, F8, FT9, FC5, FC1, FC2, FC6, FT10, T7, C3, Cz, C4, T8, TP9, CP5, CP1, CP2, CP6, TP10, P7, P3, Pz, P4, P8, O1, Oz, O2) with an actiCAP Slim 10/20 positioning system, referenced to FCz, using the BrainVision actiCHamp high-impedance system (Brain Products GmbH, Gilching, Germany). Impedances were maintained at under 17 kOhms for the duration of the recording. We acquired data at a bandpass of DC to 131 Hz, digitized at a 500 Hz sampling rate. After the acquisition, we filtered out the 60 Hz electrical noise from the continuous data using the CleanLine v2.0 toolbox plugin using a sliding window of 4 s with 50% overlap as implemented in EEGLAB [17]. This plugin adaptively estimates and removes sinusoidal noise, such as electrical line noise, from scalp channels using multi-tapering and a Thompson F-statistic [25]. In brief, the continuous data is traversed by a sliding window. Within each window, the signal is transformed to the frequency domain using a multi-taper FFT. The complex signal (i.e., amplitude and phase) was obtained for each frequency. Under the assumption of a deterministic sinusoid embedded in white noise, this plugin can regress the multi-taper transform (i.e., spectrum) of the line noise from the multi-taper spectrum of the brain signal data at a given frequency. The regression coefficient is a number representing the phase and amplitude of the deterministic sinusoid. From this, a time-domain representation of the sinusoid may be constructed and subtracted from the data to remove the line noise. We then bandpass filtered the continuous data from 0.3 to 70 Hz using the default Hamming windowed sinc FIR filter (order = 2 × highpass frequency cutoff). This was followed by re-referencing the EEG signal to the common average reference of the 32 electrode channels (i.e., the reference channel was not re-introduced as a data channel). We performed independent component analysis (ICA) on the continuous data as implemented in EEGLAB with the ‘pca’, ‘runica’, and ‘number of components = 30′ options to match the data rank for ICA for up to 30 components [26]. Components containing artifacts associated with blinks, eye movements, muscle artifacts, and remaining line noise were first classified using EEGLAB’s ICLabel classifier toolbox that utilizes crowd-sourced IC labels [27]. No components were removed until the artifact components were manually checked using the criteria described in Makeig and Onton [28] and then removed from the dataset. One incomplete subject was removed as it had insufficient data for analysis.

Trials were segmented to include the time between fixation onset and stimulus onset as baseline and the time between stimulus onset and response. Fixation and stimulus onsets, as well as response triggers, were recorded in an event list generated with ERPLAB [29]. Epochs were generated throughout the entire period from question onset to response, resulting in variable-length epochs.

For the purpose of this proof-of-concept paper, we focused on midline electrodes. Midline effects are well demonstrated in studies of working memory and general task performance, similar to what we expect to be engaged during the completion of multiple choice and script concordance questions [30,31,32].

### 2.5. Time/Frequency Analysis

Time-frequency spectrograms were created for each individual trial using the new time function as part of the EEGLAB package [17]. Hanning discrete Fourier transform (DFT) was calculated from the baseline period, i.e., onset of fixation to stimulus onset, that included a window size of 1200 samples and a maximum frequency of 40 Hz. The resulting matrices had 192 frequencies and 200 timepoints for each channel. The proportion of the trial that was used as a baseline was removed from the beginning of the timepoints, and the data were subsampled to 100 total timepoints. The final matrices of each trial were averaged for each question type for each subject. The set of 2-dimensional matrices of time by frequency for each channel, question type, and subject were used for univariate analysis.

Prior to PLS analysis, we reshaped the data to collapse time and frequency together, resulting in a 2-dimensional matrix of channels by time/frequency for each question type and subject.

### 2.6. Univariate Analysis

Multiple choice questions were compared to script concordance questions for novices and experts together using paired *t*-tests at each time/frequency and channel. Novices and experts were compared for both question types together using independent sample *t*-tests at each time/frequency and channel. FDR correction was applied to control for Type I error, with a threshold of q < 0.1.

### 2.7. Partial Least Squares Analysis

Time/frequency power data were statistically assessed with partial least squares (PLS) analysis [18,19], a multivariate approach that allowed us to identify large-scale group- and condition-dependent changes in the spatiotemporal distributions of time/frequency measures. In brief, PLS extracts latent variables (LVs) that identify patterns of similarities or differences in a brain signal measure between conditions and groups. In the most common usage of PLS, contrasts across groups or conditions are not specified in advance. Rather, the algorithm extracts LVs explaining the covariance between groups/conditions and brain signal measure in order of the amount of covariance explained with the most explained listed first. Task-related PLS begins by creating a data matrix with subjects and conditions as rows, spectral power at all measured times and frequencies for every electrode as columns. PLS uses singular value decomposition to extract LVs, which contain three vectors. The first vector consists of a singular value, which indicates the strength of the effect expressed by the LV. The remaining two vectors relate to experimental design and the brain signal measure. The experimental design vector contains design saliences, which indicate the degree to which each condition within each group is related to the time/frequency pattern identified in the LV. These design saliences can be interpreted as the contrast that codes the effect depicted in the LV. The brain signal vector contains time/frequency saliences. These are numerical electrode weights that identify the collection of electrodes and time/frequency combinations that are most related to the effects expressed in the LV. For each LV, there is one salience per electrode, time, and frequency combination that applies to all groups and all conditions. To obtain summary measures of each participant’s expression of an LV, we calculated brain scores by multiplying the vector of electrode weights by the observed value of the brain signal measure and summing over all brain signal measures for each participant. These brain scores were calculated for each condition and then mean-centered using the group mean across all conditions.

Statistical assessment in PLS is performed across two levels. First, the overall significance of each LV is assessed with permutation testing [33]. For each subject, sampling without replacement is used to reassign the order of conditions. PLS is calculated for each sample, and the number of times a singular value exceeds the observed singular value relative to the total number of permuted samples is used to assess significance. A LV was considered significant if the observed singular value exceeded the permuted singular value in more than 95% of the permutations (*p* < 0.05). Second, bootstrap resampling is used to estimate confidence intervals around electrode weights in each LV. Bootstrap samples were created by resampling subjects with replacement, and PLS was recalculated for each new sample. Distributions of bootstrapped values were used to create standard errors for electrode weights and confidence intervals for averaged brain scores, allowing for an assessment of the relative contribution of particular electrodes, times, and frequencies and the stability of the relationship with conditions and experience groups [34,35]. No corrections for multiple comparisons are necessary because the electrode saliences are calculated in a single mathematical step on the whole brain. For this paper, we chose a bootstrap ratio threshold of 2, corresponding approximately to a 95% confidence interval or a *p*-value < 0.05, to display our effects. Because our data is 2 dimensional for each channel, viewing bootstrap ratio results required separating time and frequency to reshape the data into time-by-frequency arrays for each channel.

## 3. Results

### 3.1. Varying Lengths of Trials

**Figure 2:**
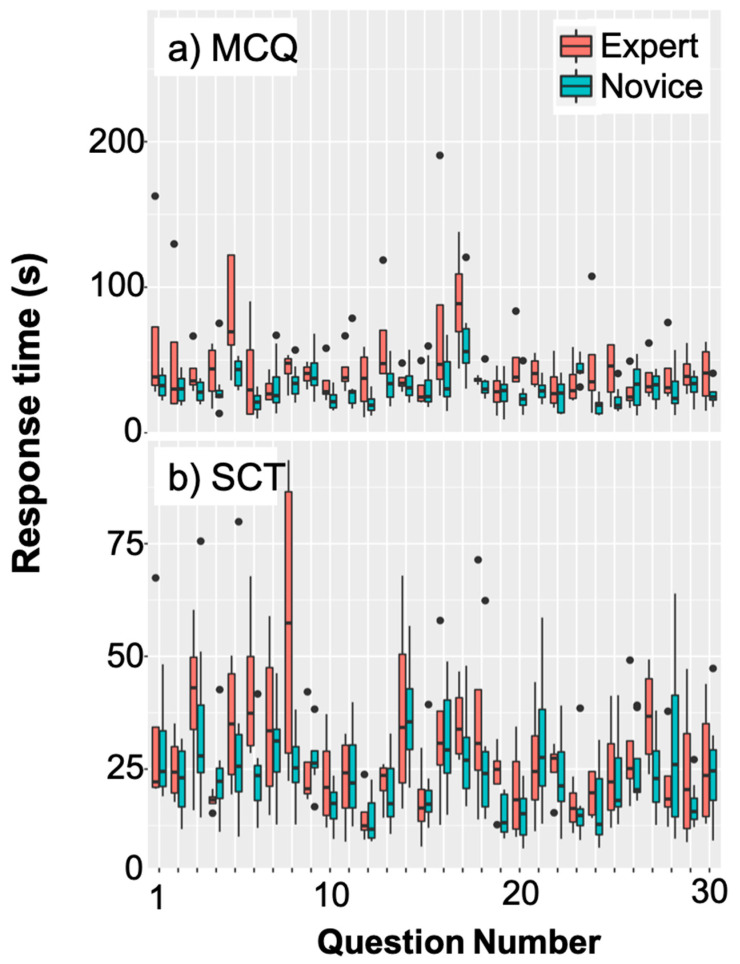
Box plot depicting the variability in length of trials based on response times (RT; in seconds) for each question in (**a**) the MCQ test condition and (**b**) the SCT condition as performed by experts (red) and novices (blue). Thick horizontal lines are the median RT, whiskers indicate the range of RTs (minimum to maximum), and the black dots indicate outlier RTs.

Figure 2 illustrates the challenge of quantifying EEG data with varying trial lengths due to the wide range of response times across test types (MCQ vs. SCT) and groups (expert vs. novice).

### 3.2. Univariate Statistics

We used paired *t*-tests of every combination of time and frequency to assess differences between reading and answering multiple choice questions and reading and answering script concordance questions in novices and experts together. No differences between script concordance and multiple-choice questions remained significant at any of the midline channels when using the false discovery rate to control for type I errors.

We used independent sample *t*-tests for every time and frequency combination to assess differences between novices and experts, with question types considered together. No differences between novices and experts remained significant at any of the midline channels when using the false discovery rate to control for type I errors.

### 3.3. PLS

**Figure 3:**
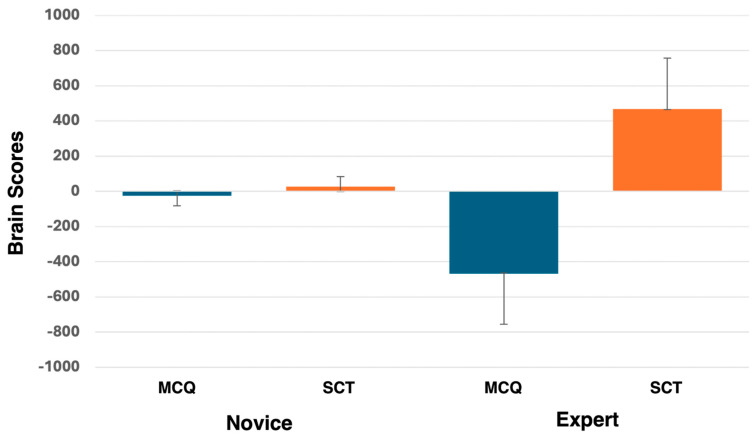
Averaged brain scores for PLS analysis comparing novice and expert time-frequency data while answering MCQ and SCT questions.

We used rotated (data-driven) task-PLS of time/frequency power values to test for effects of question type (condition) and experience level (group). One significant latent variable was extracted, showing a contrast between multiple choice and script concordance tests for experts only (*p* = 0.0020, Figure 3), potentially suggesting differences in cognitive processes accessed and used for these assessment methods.

**Figure 4:**
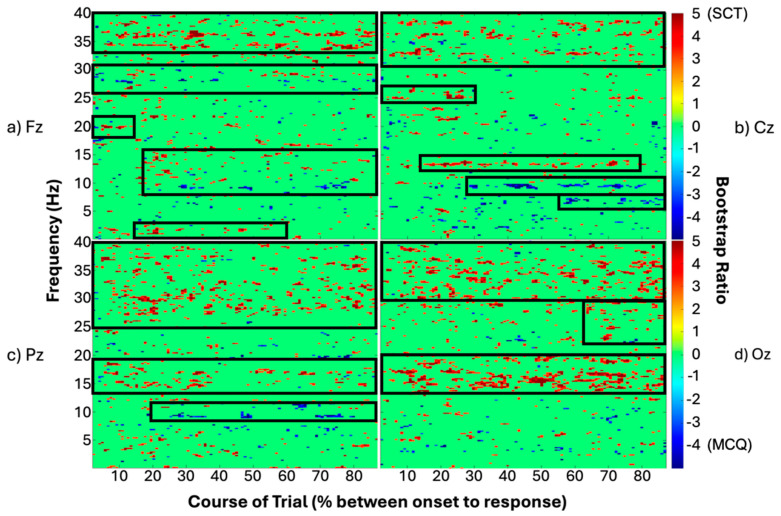
Time-frequency spectral power plots at electrode locations (**a**) Fz, (**b**) Cz, (**c**) Pz, and (**d**) Oz, respectively. Positive bootstrap ratios (>2) indicate regions that demonstrate a stable relationship between conditions (i.e., greater spectral power for SCT in experts). Negative bootstrap ratios (<−2) indicate regions that demonstrate a stable relationship between conditions (i.e., greater spectral power for MCQ in experts). The black outlined boxes highlight clusters of significant brain activity.

Output bootstrap ratio values were plotted and reshaped into time by frequency matrices for each channel. The midline frontal electrode, Fz (Figure 4a), showed higher power for SCT questions in experts in the gamma range (>35 Hz) throughout the trials. Power in the beta frequency range (~20 Hz) was higher for the experts’ SCT questions for the first 20% of the trial, and the high-alpha/low-beta frequency range (12–16 Hz) showed more power from 20% of the trial time course and onwards. Power in the theta power frequency range (~2–3 Hz) was increased for experts during SCT questions in the first 60% of the trial time course. Increased power for MCQ was observed in the high-beta frequency range (~28 Hz) for experts throughout the trial and in the alpha frequency range (~10 Hz) starting from 30% of the trial time course and throughout.

At the midline central electrode, Cz (Figure 4b), we observed higher power for experts during SCT questions in the gamma frequency range (~30+ Hz) throughout the time course of the trials. Increased power for SCT in experts was also observed in high-beta (~25 Hz) for the first 30% of the trial course and high-alpha (~13 Hz) from 15% to 80% of the trials’ time course. Increases in power for MCQs were observed in experts in the alpha frequency band (~10 Hz) starting from ~25% of the trials’ time course through to the end of the trials, and low-alpha (~8 Hz) starting from 55% of trials’ time course to the end.

At the midline parietal electrode, Pz (Figure 4c), we observed higher power for experts during SCT questions in the high-beta and gamma frequency ranges (>25 Hz) and in the high-alpha (~15–17 Hz) throughout the trials’ time course. Increased power for experts during MCQs was observed in the alpha range (~10 Hz) starting from 20% of the trials’ time course through to the end of the trials.

At the midline occipital electrode, Oz (Figure 4d), we observed higher power for experts during SCT questions in the low-beta (~15–20 Hz) and gamma frequency (>30 Hz) ranges, respectively, throughout the time course of the trials, while for high-beta (~23–30 Hz) we see higher power from 65% of the trials’ time course and onwards. There were no notable sustained changes in power for MCQ questions at this location.

## 4. Discussion

The aim of this proof-of-concept work was to determine whether we could extract consistent patterns of time-frequency power from variable epoch lengths of trials, embracing the variability of decision-making without losing data based on response time. Using univariate methods, namely multiple *t*-tests, we found no significant differences between conditions or groups that survived type I error using the false discovery rate (FDR). However, using a multivariate method, namely PLS analysis, we were able to find consistent and stable differences between conditions for one group only. The extracted latent variable reflected a different spatiotemporal pattern of activity supporting multiple choice and script concordance questions in experts only. The difference between question types was expressed in terms of frequency band (e.g., alpha at ~10 Hz) and in terms of proportions of trial completion (e.g., 10% complete). Specifically, we found typical frequency bands contributing to the contrast between question types at different electrode locations. For example, at midline Cz, power in the alpha band (~10 Hz) was found to be greater for multiple choice questions, and greater power in the gamma band (>30 Hz) was associated with script concordance questions. In terms of time, we found there are portions of the trial that more stably contribute to the contrast between question types than others. For example, at midline Cz, greater power in the gamma band for script concordance questions was found throughout the trial; however, an increase in power in the alpha band (~10 Hz) began about 20% of the way through the trials.

Comparing PLS results to the univariate findings requires caution, as these methods work very differently. While univariate analyses seek to verify a certain relationship for every timepoint and frequency, PLS results look for spatiotemporal patterns that support a certain contrast in design. However, it is undeniable that the many separate tests required for univariate statistics on this type of data, in this case 192 frequencies for each of 100 timepoints, increases the risk of type I error. In comparison, multivariate analysis considers all times and frequencies in just one test and, therefore, does not require any adjustment for type I error. In our data, we were able to extract clear patterns of power where univariate statistics using FDR correction failed to find any significance. It is understandable that such a finding might lead to skepticism, and there are a few considerations for the analysis undertaken here. Firstly, this analysis was conducted with only 4 experts and 8 novices, which leads to low power, particularly for between-subject comparisons. To verify that such a finding was not driven by one expert alone, we plotted z-scores for each expert subject for individual comparison and found that the time/frequency regions that were stable in the PLS analysis were present in all 4 expert participants. As a result, we are confident that our findings are not driven by one expert alone.

In terms of resampling the data to adjust for variance in decision-making processes, we were able to find consistent differences between question types for experts with PLS. Conducting this analysis assumes that participants will engage in similar cognitive processes at similar points in the trial. It is not necessarily the case that for every question, an individual will go through the exact same cognitive steps to arrive at an answer, and in the most extreme cases, thinking about 10% of the way through a 10 s trial is very different from 10% of a 70 s trial. However, we were able to extract a significant latent variable embracing this level of variability.

Some of the most notable spatiotemporal differences we found were in the gamma and beta frequency bands. We found increased power in gamma (~30+ Hz) that spanned the entire course of the trials at midline frontal (Fz) and central (Cz) sites. We also measured increased power in the high-beta frequency range (~20–30 Hz) that spanned the whole trial primarily at midline parietal (Pz) and occipital (Oz) sites. A previous study in monkeys found that prefrontal gamma frequency activity increased with increasing information load and that beta frequency activity is associated with top-down processes such as decision-making [36]. Furthermore, a study in humans found that the gamma frequency band is associated with the process of comparing what is in one’s memory to what is presented in a task [37], which is expected in a test-taking environment. It is possible, therefore, that the increased power in gamma frequency we found over frontal and central sites for experts completing script concordance as compared to multiple-choice questions could be related to an increase in information processing and memory comparison. The reported increase in high-beta frequency power at parietal and occipital sites for experts during script concordance, as compared to multiple-choice, could be related to an increased demand for decision-making. In a human study on decision-making, Siegel, Engel, and Donner [38] found that increased frontal-parietal low beta frequency (12–15 Hz) power predicted whether the participant would make a correct choice. Consistent with the literature, we similarly found increased high-alpha/low-beta frequency power that spanned the entire trials’ length at the midline central site associated with script concordance questions in experts. Our results are consistent with previous work that suggests this method may be a viable path forward to studying cognitive processes over variable time periods.

## 5. Limitations and Future Directions

This proof-of-concept study is meant to address ecological validity in applied decision-making studies using EEG. Granted, the primary limitation of this study is the low sample size, which relates to lack of power. The exact spatiotemporal pattern associated with this contrast may differ with a larger sample size, and for this reason, we are cautious when drawing conclusions as to the neurocognitive implications of our findings. Applying this analytical method to a larger dataset will allow more confidence in the results and potentially improve the power of multiple univariate tests to allow a more complete comparison point for the multivariate results. Another possible limitation is generalizability to other time-variable research paradigms. Here, we compare the application of the analysis to two specific question types used for the assessment of decision-making in health students and professionals. This specific application is one that, to our knowledge, has not been widely investigated with EEG or other neuroimaging techniques, and we, therefore, have little expectation of what we could find. We see this as an advantage as all work in this area is exploratory in nature, and it is possible that this resampling method could uniquely address time-varying, naturalistic challenges such as this one. Finally, we recognize that there is a difference between “brain time” and “real-time” [39]. Our study strives to capture what the brain is doing in “real-time” where some participants respond slower to an event compared to others. Here, the event is a decision-making process that includes reading, thinking, making a decision, and responding (and possibly evaluating the decision after the response, i.e., feedback), which may take some people longer than others. We used those real-time differences and created a “percentage of time duration” to allow us to directly compare the cognitive reasoning process across subjects and allow for group averages.

## 6. Conclusions

In the examination of exploring neurocognitive processes involved in authentic decision-making or test-taking situations, we have demonstrated the possibility of using resampling and PLS to allow for the use of the entirety of variable-length trials to find consistent effects. In comparison to methods that carefully construct questions to manipulate suspected cognitive processes at known periods, this method allows the researcher to utilize more naturalistic settings related to the decision-making environment. This methodology could expand the current understanding of neurocognition in authentic decision-making teaching and learning scenarios, as well as other fields.

## Data Availability

The datasets analyzed during the current study are not publicly available due to confidentiality issues of the small sample, which is part of a larger dataset, but are available from the corresponding author upon reasonable request.

## References

[1] Gold J.I., Shadlen M.N. (2007). The neural basis of decision-making. Annu. Rev. Neurosci..

[2] Reder S. (1987). Strategy selection and question answering. Cogn. Psychol..

[3] Boudewyn M., Luck S.J., Farrens J.L., Kappenman E.S. (2017). How many trials does it take to get a significant ERP effect? It depends. Psychophysiology.

[4] Deco G., Jirsa V., McIntosh A.R., Sporns O., Kotter R. (2009). Key role of coupling, delay, and noise in resting brain fluctuations. Proc. Natl. Acad. Sci. USA.

[5] Onton J., Delorme A., Makeig S. (2005). Frontal midline EEG dynamics during working memory. NeuroImage.

[6] Jacobs J., Hwang G., Curran T., Kahana M.J. (2006). EEG oscillations and recognition memory: Theta correlates of memory retrieval and decision-making. NeuroImage.

[7] Ahmed M.Z.I., Sinha N., Ghaderpour E., Phadikar S., Ghosh R. (2023). A novel baseline removal paradigm for subject-independent features in emotion classification using EEG. Bioengineering.

[8] Erat K., Sahin E.B., Dogan F., Merdanoglu N., Akcakaya A., Durdu P.O. (2024). Emotion recognition with EEG-based brain-computer interfaces: A systematic literature review. Multimed. Tools Appl..

[9] Picton T.W. (1992). The P300 wave of the human event-related potential. J. Clin. Neurophys..

[10] Proudfit G.H. (2012). The reward positivity: From basic research on reward to a biomarker for depression. Psychophysiology.

[11] Pfurtscheller G., Aranibar A. (1979). Evaluation of event-related desynchronization (ERD) preceding and following voluntary self-paced movement. Electroencephalogr. Clin. Neurophysiol..

[12] Bressler S.L., Freeman W.J. (1980). Frequency analysis of olfactory system EEG in cat, rabbit, and rat. Electroencephalogr. Clin. Neurophysiol..

[13] Makeig S. (1993). Auditory event-related dynamics of the EEG spectrum and effects of exposure to tones. Electroencephalogr. Clin. Neurophysiol..

[14] Neuenschwander S., Varela F.J. (1993). Visually triggered neuronal oscillations in the pigeon: An autocorrelation study of tectal activity. Eur. J. Neurosci..

[15] Weiss S., Rappelsberger P. (1996). EEG coherence within the 13-18 Hz band as a correlate of a distinct lexical organisation of concrete and abstract nouns in humans. Neurosci. Lett..

[16] Tallon-Baudry C., Bertrand O., Delpuech C., Pernier J. (1997). Oscillatory gamma-band (30–70 Hz) activity induced by a visual search task in humans. J. Neurosci..

[17] Delorme A., Makeig S. (2004). EEGLAB: An open source toolbox for analysis of single-trial EEG dynamics including independent component analysis. J. Neurosci. Methods.

[18] Lobaugh N.J., West R., McIntosh A.R. (2001). Spatiotemporal analysis of experimental differences in event-related potential data with partial least squares. Psychophysiology.

[19] McIntosh A.R., Lobaugh N.J. (2004). Partial least squares analysis of neuroimaging data: Applications and advances. NeuroImage.

[20] Kovacevic N., McIntosh A.R. (2003). Groupwise independent component decomposition of EEG data and partial least squares analysis. NeuroImage.

[21] McIntosh A.R., Kovacevic N., Itier R.J. (2008). Increased brain signal variability accompanies lower behavioral variability in development. PLoS Comput Biol..

[22] Szostakiwskyj J.M.H., Willatt S.E., Cortese F., Protzner A.B. (2017). The modulation of EEG variability between internally- and externally-driven cognitive states varies with maturation and task performance. PLoS ONE.

[23] Charlin B., Roy L., Brailovsky C., Goulet F., van der Vleuten C. (2000). The Script Concordance Test: A tool to assess the reflective clinician. Teach. Learn. Med..

[24] Gawad N., Wood T.J., Cowley L., Raiche I. (2021). How do cognitive processes influence script concordance test responses?. Med. Educ..

[25] Mitra P., Bokil H. (2007). Observed Brain Dynamics, Chapter 7.3.4.

[26] Makeig S., Bell A., Jung T.P., Sejnowski T.J., Touretzky D.S., Mozer M., Hasselmo M.E. (1996). Independent component analysis of electroencephalographic data. Advances in Neural Information Processing Systems 8.

[27] Pion-Tonachini L., Kreutz-Delgado K., Makeig S. (2019). ICLabel: An automated electroencephalographic independent component classifier, dataset, and website. NeuroImage.

[28] Makeig S., Onton J., Luck S.J., Kappenman E.S. (2013). ERP features and EEG dynamics: An ICA perspective. Oxford Handbook of Event-Related Potential Components.

[29] Lopez-Caleron J., Luck S.J. (2014). ERPLAB: An open-source toolbox for the analysis of event-related potentials. Front. Hum. Neurosci..

[30] Hsieh L.-T., Ranganath C. (2014). Frontal midline theta oscillations during working memory maintenance and episodic encoding and retrieval. NeuroImage.

[31] Meyer L., Grigutsch M., Schmuck N., Gaston P., Friederici A.D. (2015). Frontal–posterior theta oscillations reflect memory retrieval during sentence comprehension. Cortex.

[32] Sauseng P., Klimesch W., Schabus M., Doppelmayr M. (2015). Fronto-parietal EEG coherence in theta and upper alpha reflect central executive functions of working memory. Int. J. Psychophysiol..

[33] Good P. (2000). Dependence. Permutation Tests.

[34] Efron B., Tibshirani R. (1986). Bootstrap methods for standard errors, confidence intervals, and other measures of statistical accuracy. Stat. Sci..

[35] Efron B., Tibshirani R.J. (1994). An Introduction to the Bootstrap.

[36] Kornblith S., Buschman T.J., Miller E.K. (2015). Stimulus load and oscillatory activity in higher cortex. Cereb. Cortex.

[37] Herrmann C.S., Munk M.H.J., Engel A.K. (2004). Cognitive functions of gamma-band activity: Memory match and utilization. TiCS.

[38] Siegel M., Engel A.K., Donner T.H. (2011). Cortical network dynamics of perceptual decision-making in the human brain. Front. Hum. Neurosci..

[39] van Bree S., Melcon M., Kolibius L.D., Kerren C., Wimber M., Hanslmayr S. (2002). The brain time toolbox, a software library to retune electrophysiology data to brain dynamics. Nat. Hum. Behav..

